# An Unusual Destination: Neuroendocrine Carcinoma Arising in the Breast, an Uncommon and Aggressive Entity

**DOI:** 10.7759/cureus.109028

**Published:** 2026-05-17

**Authors:** Hanan Bailal, Fadoua Jebrouni, Kaouthar Khater, Asmae Bali, Hind Chibani, Ouissam Al Jarroudi, Sami Aziz Brahmi, Said Afqir

**Affiliations:** 1 Medical Oncology, Mohammed VI University Hospital, Faculty of Medicine and Pharmacy of Oujda, Mohammed First University of Oujda, Oujda, MAR

**Keywords:** breast, carcinoma, metastasis, neuroendocrine, platinum

## Abstract

Neuroendocrine-differentiated breast carcinoma is an uncommon and challenging subtype of breast cancer, with aggressive clinical behavior. Owing to the limited number of reported cases and the lack of standardized treatment guidelines, its clinical presentation, natural history, and optimal management remain poorly defined, particularly in the metastatic setting. Herein, we report the case of a 66-year-old woman who was referred to our department for the management of a de novo metastatic small-cell neuroendocrine carcinoma of the breast.

## Introduction

Neuroendocrine carcinomas are rare tumors, accounting for 0.1% to 5% of breast cancers and less than 1% of neuroendocrine neoplasms [1]. Neuroendocrine differentiation within breast cancer was first described in 1963, according to Feyrter and Hartmann [2]. But the first instance of primary neuroendocrine breast carcinoma was not reported until 1977, when Cubilla and Woodruff proposed a clinical and histological categorization for this uncommon subtype of breast cancer [3]. They are characterized by neuroendocrine architectural and cytological features, which must be confirmed by positive immunohistochemistry for neuroendocrine markers (such as chromogranin and synaptophysin) [4]. Due to their rarity, the data on breast carcinomas that are now accessible are mostly based on tiny retrospective series or single observations. As a result, there is an ongoing discussion in the literature over their definition, histopathological diagnostic standards, and treatment approaches.

We describe the case of a 66-year-old female patient who was admitted to our department to be treated for a small-cell neuroendocrine carcinoma of the breast that had already spread to the liver and bones. Through this case report, we highlight the clinical, diagnostic, and therapeutic features of this uncommon subtype of breast cancer.

## Case presentation

The patient, a 66-year-old woman with a family history of colorectal cancer, especially in her brother, was diagnosed with hypertension five years ago. She is postmenopausal. The patient sought medical assistance after seeing an expansion of a left axillary lymph node. She had a breast ultrasound, which verified the existence of a left axillary lesion classed as Breast Imaging Reporting and Data System (BIRADS) 4A but ruled out the occurrence of breast lesions in both breasts. This lesion was subsequently biopsied.

The histopathology results confirmed the presence of a poorly differentiated, infiltrating carcinomatous process (Figures 1, 2) while immunohistochemistry verified the diagnosis of a poorly differentiated small-cell neuroendocrine carcinoma by showing positivity for neuroendocrine markers like synaptophysin and Insm 1, chromogranin A, and p40 were negative, and the Ki-67 proliferation index was high, estimated at 70%.

**Figure 1:**
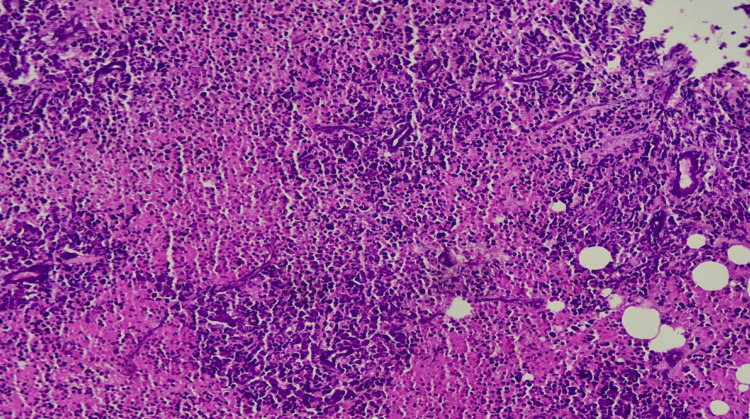
Poorly differentiated, infiltrating cells arranged in solid nests, with moderate cytoplasm, large hyperchromatic nuclei, and marked pleomorphism, associated with high mitotic activity. Hematoxylin and eosin (H&E) x10

**Figure 2:**
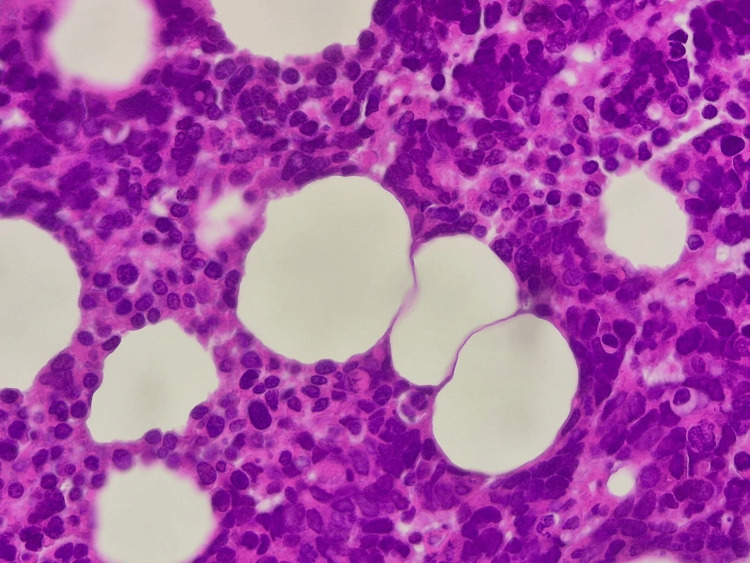
A diffuse, infiltrating tumor consisting of small, atypical cells with scant cytoplasm and a high nuclear-to-cytoplasmic ratio, arranged in solid nests within the breast parenchyma. Hematoxylin and eosin (H&E) x40

Then a PET scan was done, and the results showed that the carcinoma was aggressive, with substantial bone involvement in the axial and extra-axial skeleton and evidence of liver metastases, but no potentially fatal lesions (Figures 3, 4).

**Figure 3:**
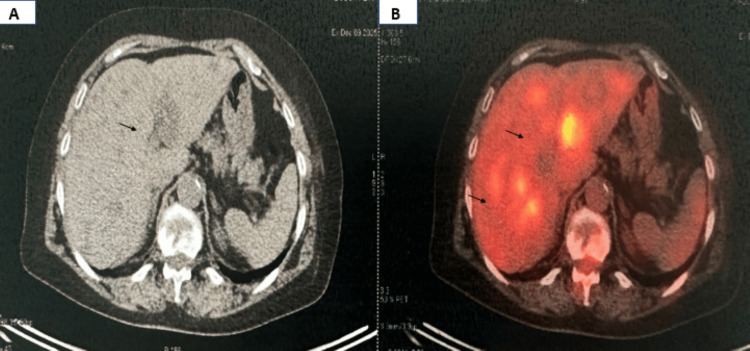
(A, B) PET scan images showing liver lesions that appear to be secondary. The black arrows indicate hypermetabolic foci corresponding to hepatic metastases in segments V and VI.

**Figure 4:**
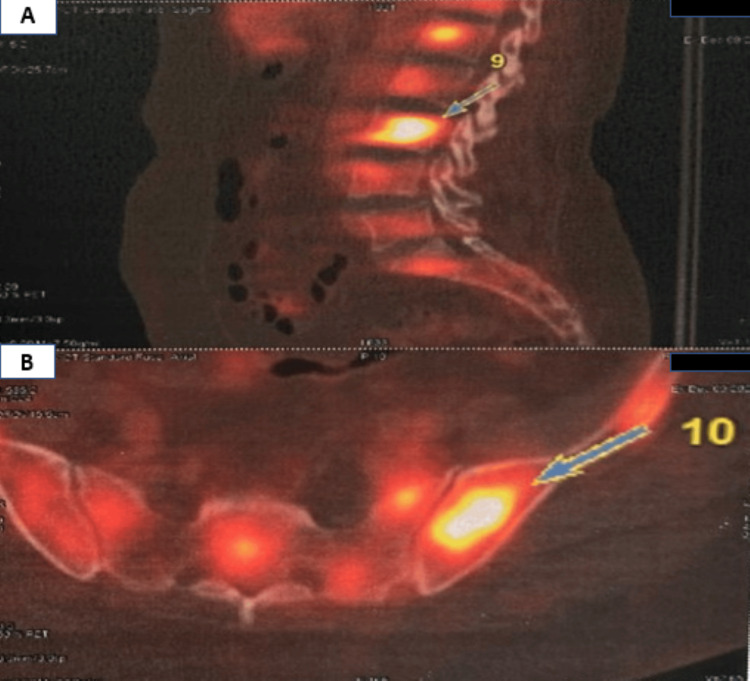
PET scan images showing active secondary bone lesions in the spine (A) and outside the spinal canal (B) (lesions shown by arrows 9 and 10).

First-line platinum-based chemotherapy was initiated for this patient, who had good clinical tolerability during the first cycle.

## Discussion

Neuroendocrine carcinomas are rare tumors, accounting for 0.1% to 5% of breast cancers [1]. Over the past two decades, the classification of neuroendocrine neoplasms has evolved, leading to a division into two main categories: well-differentiated neuroendocrine tumors (NETs) and poorly differentiated neuroendocrine carcinomas (NECs) [1].

Compared to other common breast tumors, the diagnostic approach begins with standard radiological assessment, including mammography and ultrasound-guided biopsy. However, the definitive diagnosis relies primarily on histological examination, supported by immunohistochemical analysis [5].

As a result, the tumor's histology may show an organoid, trabecular, or sheet-like architecture, with comparatively homogeneous cells that have rounded nuclei with fine chromatin and mild cytoplasm. The tumor cells are tiny, have little cytoplasm, and have a high mitotic index in some aggressive forms, including small-cell neuroendocrine carcinoma of the breast [4].

Confirming the diagnosis is mostly dependent on immunohistochemical analysis. Neuroendocrine markers like chromogranin A and synaptophysin are frequently expressed by tumor cells, and the proliferation index, measured by Ki-67, aids in determining the aggressiveness of the tumor.

Additionally, the expression of markers specific to the breast, like GATA3 or mammaglobin, helps clarify that the tumor originated in the breast and rules out metastasis from an extra-mammary neuroendocrine tumor, especially one that originated in the gastrointestinal tract or lung. Most breast neuroendocrine carcinomas are of the luminal type at the molecular level [6]. However, neuroendocrine differentiation seems to be related to higher aggressiveness, even though this profile is typically associated with a better prognosis in conventional carcinomas. Ki-67 is a key prognostic marker, and a high Ki-67 index is frequently associated with rapid metastatic progression, particularly to the liver, bones, and lungs. This is illustrated in our case where the Ki-67 index was estimated at 70%, indicating highly aggressive tumor behavior [7].

Due to the lack of an international consensus and the limited prevalence of these breast tumors, the management of breast carcinomas is multimodal. The mainstay of treatment is surgery, which combines radical mastectomy with lymph node dissection or breast-conserving surgery [8].

Adjuvant therapy recommendations are largely in line with conventional clinical practice guidelines for breast cancer. In metastatic disease, the standard first-line treatment has traditionally been the platinum-etoposide combination, based on the strategy used for small-cell lung cancer, despite the fact that this regimen produces significant initial objective response rates, ranging from 40% to 70%. The length of response is typically brief, with a median progression-free survival of approximately four to six months [9].

Somatostatin analogs may be helpful for cancers that express somatostatin receptors, especially when combined with hormone therapy [10]. Regarding the role of immunotherapy, the available literature is limited. The biological features of this tumor, however, point to minimal immunogenicity, low mutational burden, and low sensitivity to immunotherapy as a monotherapy [11].

Nevertheless, immunotherapy may be a potential alternative in poorly differentiated or high-grade forms, especially those that resemble small-cell neuroendocrine carcinomas, especially when paired with chemotherapy. This strategy is based on research on small-cell lung cancer; however, there isn't yet any solid evidence that specifically addresses breast cancer [8].

There is an ongoing debate over the prognosis for neuroendocrine-differentiated breast cancers. It is widely acknowledged that patients with neuroendocrine carcinomas have a worse prognosis and a shorter overall survival than those with invasive breast carcinomas [12]. Small-cell and large-cell neuroendocrine breast carcinomas appear to have comparable results, according to some data, but small-cell neuroendocrine breast carcinoma had the worst prognosis, according to another study [8].

## Conclusions

Neuroendocrine-differentiated breast carcinoma is a rare and heterogeneous entity, for which management guidelines remain undefined due to the limited evidence available. Through this case of metastatic disease, we highlight the often aggressive nature of these tumors, characterized by a high capacity for dissemination and a generally poor prognosis, particularly in the presence of a high proliferation index.

The lack of specific recommendations generally leads to the extrapolation of treatment strategies from conventional breast carcinomas or neuroendocrine tumors in other sites. However, this empirical approach has its limitations, particularly in advanced stages. The emergence of targeted therapies and immunotherapy offers promising prospects, although their role in this tumor subtype remains to be defined.
